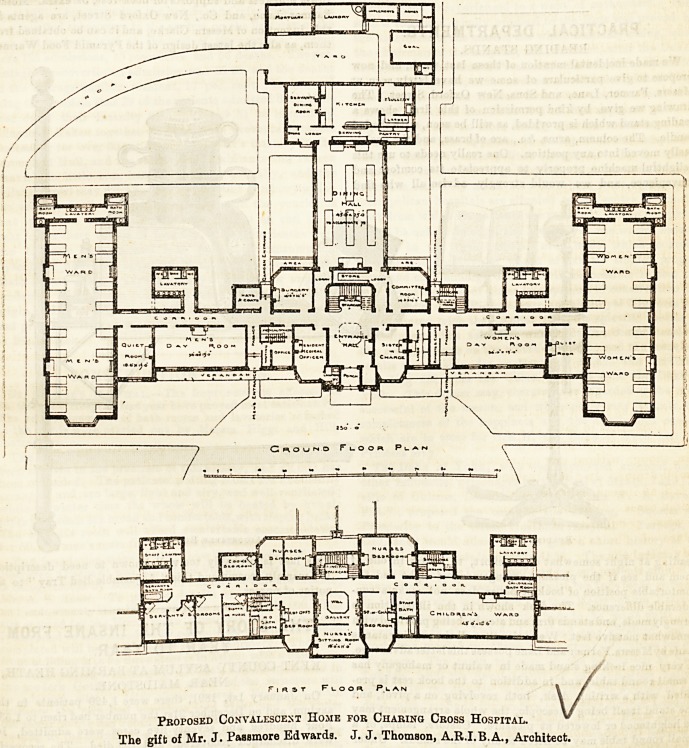# Convalescent Home for Charing Cross Hospital

**Published:** 1893-05-27

**Authors:** 


### The Institutional Workshop.

### WITHIN THE HOSPITALS.

### CONVALESCENT HOME FOR CHARING CROSS HOSPITAL.

The fact that Charing Cross Hospital is to possess a convalescent home of its own is due to the munificence of Mr. J. Passmore Edwards, who offered to build and furnish at his own proper cost a convalescent home for fifty patients for the provision is difficult to see. Then, again, if cases of a more acute nature than ordinary convalescents are to be treated here the size of the wards is far too great. The wards are too large for convalescents even if double the number of patients were to be accommodated, but to put all the male patients into one ward and all the female patients into another is a grave error, as it puts any classification of patients quite out of question. The provision of water-closets for patients is

Proposed Convalescent Home for Charing Cross Hospital.

The gift of Mr. J. Passmore Edwards. J. J. Thomson, A.R.I.B.A., Architect.

sole use of the hospital. The number of beds would seem somewhat out of proportion to the size of the hospital for which the home is to be built, unless it is the intention of the authorities to use it rather as a semi-convalescent home than as a convalescent home in the ordinary sense of the term. Possibly some such intention as this may account for the unusual provision of rooms for a resident medical officer, otherwise the necessity, or, indeed, the wisdom, of such a very inadequate, and should be at least doubled, and urinal accommodation for the men entered from the outside should also be provided. The children's ward is on the upper floor, but there does not seem to be any provision for a day-room for them. This is at least as important for children as it is for adults.

The dining hall is planned to accommodate 72 patients, thus providing for increased wards in the future. The kitchen offices adjoin the dining hall, and certainly do not err on the side of extravagance. The larder is too small, and its approach through the scullery is a most objectionable arrangement. On one side of the courtyard at the back of the kitchen offices is the mortuary, its door being in full view of the kitchen windows; adjoining this is a laundry, about equal in size to the requirements of a small cottage hospital, and other offices.

The plans appear to us ill-considered and badly arranged, and the whole scheme would be better for a thorough revision of all the parts carefully worked out in the light of practical experience.

## Figures and Tables

**Figure f1:**